# Enhanced Feedback-Related Negativity in Alzheimer’s Disease

**DOI:** 10.3389/fnhum.2017.00179

**Published:** 2017-04-28

**Authors:** Eri Nitta, Keiichi Onoda, Fuminori Ishitobi, Ryota Okazaki, Seiji Mishima, Atsushi Nagai, Shuhei Yamaguchi

**Affiliations:** ^1^Central Clinical Laboratory, Shimane University HospitalIzumo, Japan; ^2^Department of Neurology, Shimane University Faculty of MedicineIzumo, Japan

**Keywords:** Alzheimer’s disease, monitoring systems, feedback-related negativity, depression, aging

## Abstract

Alzheimer’s disease (AD), the most common cause of dementia in the elderly, results in the impairment of executive function, including that of performance monitoring. Feedback-related negativity (FRN) is an electrophysiological measure reflecting the activity of this monitoring system via feedback signals, and is generated from the anterior cingulate cortex. However, there have been no reports on FRN in AD. Based on prior aging studies, we hypothesized that FRN would decrease in AD patients. To assess this, FRN was measured in healthy individuals and those with AD during a simple gambling task involving positive and negative feedback stimuli. Contrary to our hypothesis, FRN amplitude increased in AD patients, compared with the healthy elderly. We speculate that this may reflect the existence of a compensatory mechanism against the decline in executive function. Also, there was a significant association between FRN amplitude and depression scores in AD, and the FRN amplitude tended to increase insomuch as the Self-rating Depression Scale (SDS) was higher. This result suggests the existence of a negative bias in the affective state in AD. Thus, the impaired functioning monitoring system in AD is a more complex phenomenon than we thought.

## Introduction

Executive function is a set of cognitive processes including attentional control, working memory and planning, and so on, and is necessary for selecting and successfully monitoring behavior (Alvarez and Emory, 2006; Chan et al., 2008; Diamond, 2013). Executive function is impaired in individuals with dementia, including those suffering from Alzheimer’s Disease (AD) (Collette et al., 1999; Perry and Hodges, 1999). Monitoring is a part of the executive function, and is the ability to monitor one’s own actions and responses during task performance in order to detect and correct errors. Impairment of executive function in AD patients is involved in the failure of self-monitoring (Ott et al., 1996), which may underlie subtle difficulties in coping with daily activities (Perry and Hodges, 1999). It has been suggested that deficits in self-monitoring occur after damage to the frontal lobes and other cerebral areas (Ott et al., 1996).

Feedback-Related Negativity (FRN) is a neurophysiological index that reflects the monitoring process associated with feedback inputs. FRN has been shown to be elicited by feedback stimuli (particularly negative stimuli) in a gambling task (Gehring, 2002; Hajcak et al., 2006; Holroyd et al., 2006) and a time production task (Miltner et al., 1997; Wild-Wall et al., 2009; Becker et al., 2014). This negative potential appears at a latency of 200–300 ms after feedback (Miltner et al., 1997; Falkenstein et al., 2000; Gehring, 2002; Holroyd and Coles, 2002; Nieuwenhuis et al., 2004), and is primarily distributed over the frontal-central scalp area. Generally, FRN means the negative peak obtained by subtracting the waveform for the positive feedback from that for the negative feedback, or the negative waveform obtained from the negative feedback. A great amount of evidence indicates that FRN is generated in the anterior cingulate cortex (Holroyd and Coles, 2002; Hajcak et al., 2007; Bellebaum and Daum, 2008; Holroyd et al., 2009). FRN codes negative prediction errors in the context of varying reword probabilities and magnitudes (Cohen et al., 2007; Bellebaum and Daum, 2008; Wu and Zhou, 2009; Bellebaum et al., 2010). As with other components of event-related evoked potentials (ERPs), many studies have revealed that FRN amplitude is reduced and its latency prolonged in the elderly compared to that in young individuals (Eppinger et al., 2008; Mathewson et al., 2008; Wild-Wall et al., 2009; Hämmerer et al., 2011; Pietschmann et al., 2011a), although some studies show no such age differences in FRN (Bellebaum et al., 2011). However, in line with the FRN aging effects, AD patients might show alterations of FRN. However, no reports have investigated the changes in FRN in AD patients.

Error-Related Negativity (ERN) is an ERP component elicited by an individual’s own behavioral errors. ERN measures may also provide insights to the monitoring system. Some reports have demonstrated that ERN amplitude decreases in older participants (Gehring and Knight, 2000; Falkenstein et al., 2001; Nieuwenhuis et al., 2002; Pietschmann et al., 2011a,b; Schreiber et al., 2011; Endrass et al., 2012), and is further reduced in AD patients with prolonged latency (Mathalon et al., 2003). Considering its similarity to ERN, this evidence suggests that FRN should also demonstrate decreased amplitude and delayed latency in AD patients.

P300 is a positive component at the dominantly parietal area approximately 300 ms after stimulus onset (Sutton et al., 1965), and is elicited typically by an oddball task, i.e., auditory, visual, olfactory or somatosensory stimuli (Yamaguchi and Knight, 1991a,b; Frodl et al., 2002; Polich, 2004; Bennys et al., 2007; Golob et al., 2007). This component reflects various cognitive processes, including context updating (Donchin, 1981; Polich, 2007), resource allocation (Wickens et al., 1983; Kramer et al., 1985; Polich, 2007), memory encoding (Karis et al., 1984; Fabiani et al., 1990; Johnson, 1995; Polich, 2007) and attention, stimulus evaluation, judgment and decision-making (Becker and Shapiro, 1980; Duncan-Johnson and Donchin, 1982; Kramer and Strayer, 1988; Katada et al., 2004; Gironell et al., 2005). There are many regions in the brain, especially in the parietal, temporal, prefrontal cortex and hippocampus, that contribute to its generation (Yamaguchi and Knight, 1991a; Halgren et al., 1998; Tarkka and Stokic, 1998; Kirino et al., 2000; Kiehl et al., 2001). P300 amplitude and latency are modulated by a variety of factors, including subjective probability of stimuli, stimulus saliency, availability of attentional resources (Kutas et al., 1977; Polich, 1986; Kramer and Strayer, 1988; Gonsalvez and Polich, 2002), and memory performance (Fabiani et al., 1990; Johnson, 1995). P300 has been proposed as one of the electrophysiological biomarkers of dementia (Olichney et al., 2011; Howe et al., 2014), and its usefulness has been well documented in the early diagnosis of dementia (Polich et al., 1990; Juckel et al., 2008; Ahiskali et al., 2009; Chapman et al., 2011; Vecchio and Määttä, 2011). Elderly people show a decreased amplitude and prolonged latency of P300, and these changes are more pronounced in dementia (Polich and Corey-Bloom, 2005; Bennys et al., 2007; Lai et al., 2010; Parra et al., 2012). We speculate that these findings regarding P300 may also be applicable to feedback stimuli.

Previous studies have also claimed that FRN and P300 encode different aspects of outcome evaluations (Yeung and Sanfey, 2004). These suggest that outcome evaluation can be roughly divided into two related processes: one is an early evaluation of the cognitive or motivational significance of feedback stimuli, which relates to FRN; and the other is a more elaborative evaluation of feedback stimuli, which is affected by the allocation of attentional resources such as intentionality or expectancy and is related to P300. Furthermore, other research has reported that FRN is sensitive not only to reward valence and magnitude, but also to expectancy towards reward magnitude. While P300 is sensitive to both feedback valence and reward magnitude, this sensitivity can be modulated by expectancy towards reward magnitude (Wu and Zhou, 2009). These findings suggest that FRN may play a role as a general mechanism that evaluates whether an outcome is consistent or inconsistent with expectations; whereas P300 is sensitive to the later, top-down controlled process of outcome evaluation, which links with the allocation of attentional resources, including reward valence, reward magnitude and magnitude expectancy. In AD patients, these processes of outcome evaluation for feedback stimulus are considered to be impaired.

Accordingly, we investigated the monitoring system of AD patients using a neurophysiological measure (i.e., FRN), in a gambling task. We hypothesized that, compared with healthy elderly people and healthy young people, the amplitude of FRN would be reduced, and latency delayed in AD patients due to disruption of feedback processing.

## Materials and Methods

### Participants

Twenty-four patients with (AD; 15 males, 9 females, age range from 66 to 75, mean age = 71.5, SD = 2.8), 20 healthy older subjects (HO; 13 males, 10 females, age range from 62 to 79, mean age = 69.6, SD = 6.0), and 19 healthy young subjects (HY; 10 males, 9 females, age range from 19 to 28, mean age = 22.2, SD = 2.2) participated in this study. The AD patients met the National Institute of Neurological and Communicative Disorders and Stroke and the AD and Related Disorders Association (NINCDS/ADRDA) criteria for individuals with AD. Their scores on the Mini-Mental State Examination (MMSE) were 19.3 ± 3.9, and on the Clinical Dementia Rating (CDR) were 1.1 ± 0.4, (21 patients for CDR 1, and 3 patients for CDR 2). All participants had normal or corrected-to-normal vision. Virtually all participants were right-handed, with three exceptions (one in each group). Participants in the HO and HY groups had no history of neurological or psychiatric diseases. Finally, this study was approved by the ethics committee of the Shimane University. All participants provided signed informed consent following our explanation of the study’s purpose and protocols.

### Neuropsychological Assessment

AD and HO participants were assessed using neuropsychological test batteries that included the Mini Mental State Examination (MMSE; Folstein et al., 1975), the Frontal Assessment Battery (FAB; Dubois et al., 2000), the Word Fluency Test (WFT; vegetable for the semantic category), the Self-rating Depression Scale (SDS; Zung, 1965), and the Apathy Scale (AS; Okada et al., 1998). These assessments were conducted by a trained clinical researcher within the 2-week period before the ERP experiment.

### Task, Stimuli and Procedure

**Figure 1:**
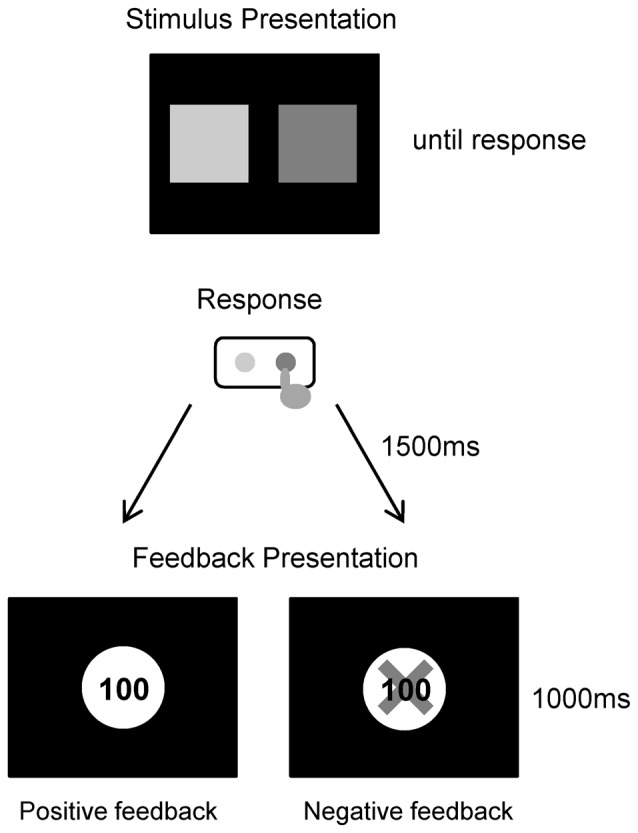
**Trial procedure for the gambling task**.

In the experiment conducted for this study, each participant performed a simple gambling task (Figure 1). Participants were comfortably seated approximately 1.5 m in front of a computer screen in an electrically shielded and sound-attenuated room. At the start of each trial, a depiction of the choice display was presented on a screen, which lasted until a participant made a response. The picture consisted of two colored squares with green and purple presented on the left and right screen sides respectively, and the relationship between the color and position was consistently maintained for all individuals. We instructed participants that they could win money by correctly choosing one of the squares. Participants then chose one of the two squares by pressing one of two corresponding buttons (i.e., left or right). A feedback stimulus appeared 1.5 s after the choice picture was turned off, and lasted for 1 s. The feedback stimulus consisted of a display of a win or loss. If the selected box was a win, a 100 yen (about 1 USD) coin appeared in the center of the screen. If the selected box was a loss, a 100 yen coin was presented with a superimposed red X. The next trial started following an inter-trial interval of 2–3 s. The experiment consisted of 120 trials (two blocks of 60 trials each). The probabilities of winning and losing for each option was equal (50%), and this was same in all three groups. Participants were told that they would be participating in a virtual game; they were instructed to try to maximize their monetary rewards. Participants performed 10 practice trials before the experiment. Total task duration including 2 blocks and rest was about 15–20 min, and the whole experiment including the installation and removal of electrodes lasted about 1 h.

### Electroencephalography (EEG) Recording and Data Reduction

The electroencephalogram (EEG) was obtained from 21 Ag/AgCl electrodes at the positions of the International 10–20 System: midline (FPz, Fz, Cz, Pz, Oz); frontal (Fp1, Fp2, F3, F4, F7, F8); central (C3, C4); temporal (T3, T4, T5, T6); parietal (P3, P4); occipital (O1, O2); and was referenced to the linked mastoids. Horizontal and vertical electrooculograms (EOG) were recorded at sites lateral to the left and right outer canthi, and above and below each eye. Impedances were kept below 5 kΩ for each electrode. The EEG signals were recorded using the BrainAmp amplifier (Brain Products, Munich, Germany) with appropriate software. The EEG signals were recorded continuously with a band pass set at 0.01–250 Hz, and a sampling frequency of 500 Hz. In addition, each participant’s reaction time (RT) was measured simultaneously with the EEG recording.

### Event-Related Potentials (ERPs)

EEG data were analyzed off-line using the BrainVision Analyzer 2 software (Brain Products, Munich, Germany). An independent component analysis (ICA) was performed on single-subject EEG data in order to correct for blink artifacts. All segments exceeding ± 100 μV were rejected as artifacts. EEG epochs were extracted beginning 200 ms before and ending 800 ms after the presentation of feedback for win and loss conditions, separately. A baseline was set at the duration of 200 ms prior to feedback stimulus onset. ERP peak amplitude and latency were derived from each individual’s average waveform. The FRN was semi-automatically measured as the most negative peak within the time window of 150–400 ms after feedback presentation, and was finally identified by visual inspection. P300 was measured as the most positive peak within the time window of 300–600 ms in the same way. The additional FRN measures were computed for each participant by subtracting the waveform after positive feedback from that after negative feedback. The peak amplitudes and latencies of the components were derived from the resulting difference wave within the time window of 150–450 ms.

### Statistics

We conducted *t*-tests on the demographic (except for the *χ*^2^ test for sex) and neuropsychological data to allow comparison of AD and HO. To check each participant’s understanding of the task, we calculated switching response ratios following negative and positive feedback, respectively. A higher switching ratio for negative feedback than for positive feedback means that participants had a tendency to avoid the option with the last negative feedback, and were able to understand the gambling task. Therefore, we compared the switching ratios between preceding negative and positive feedback using paired *t*-tests in each group. A one-way analysis of variance (ANOVA) was used to analyze the RT data, and a two-way repeated measures ANOVA (group × channel, or group × feedback condition) was performed (separately) for the amplitudes and latency of ERP components. The statistical criterion was set at a *p* value of less than 0.05, and Tukey method analysis was used for *post hoc* tests. Partial correlation analyses were also conducted to examine the relationships between the ERP components and the neuropsychological data.

## Results

### Neuropsychological and Behavioral Data

The background, neuropsychological and behavior data are summarized in Table 1. There were no significant differences in age and gender ratios between AD and HO. However, independent *t* tests revealed that there were significant differences between AD and HO on the cognitive function scores (MMSE, FAB, WFT), and that AD showed reduced cognitive function compared to HO (*t*s (45) > 5.0, *p*s < 0.001). However, affective function scores did not differ between those groups (*t*s (45) < 1.9, *p*s > 0.068). RT in the gambling task was delayed significantly in AD and HO compared to that in HY (*p*s < 0.001). Switching response ratio was higher for the following negative feedback than positive feedback in every three groups (*p*s < 0.05).

**Table 1:** **Participants’ background, neuropsychological data and behavioral data**.

	AD	HO	HY	*p*
*N*	24	25	19	
Age (years)	71.5 ± 2.8	69.6 ± 6.0	22.0 ± 2.2	0.181
Sex (M/F)	15/9	13/10	10/9	0.684
MMSE	19.3 ± 3.9	28.9 ± 1.9	–	<0.001
FAB	10.8 ± 3.4	16.0 ± 1.1	–	<0.001
WFT	8.7 ± 4.8	15.1 ± 3.6	–	<0.001
SDS	32.5 ± 7.4	30.7 ± 6.5	–	0.357
AS	12.7 ± 8.3	9.0 ± 4.7	–	0.068
RT (ms)	1884 ± 834	1027 ± 319	679 ± 395	<0.001
SR ratio following NF	0.60 ± 0.18	0.67 ± 0.15	0.56 ± 0.17	<0.001
SR ratio following PF	0.37 ± 0.20	0.24 ± 0.11	0.38 ± 0.22

*Data are shown in mean and standard deviation format. All *p*-values except for SR ratio indicate results of the comparisons between Alzheimer’s disease (AD) and healthy old (HO). The *p*-value of the SR ratio shows results of the comparisons between following Negative feedback and following Positive feedback in all groups. HY means healthy young. MMSE, Mini Mental State Examination; FAB, Frontal Assessment Battery; WFT, Word Fluency Test; SDS, Self-rating Depression Scale; AS, Apathy Scale; RT, Reaction time in gambling task; SR ratio, Switching Response ratio in gambling task; NF, Negative feedback; PF, Positive feedback*.

### ERP Waveforms

**Figure 2:**
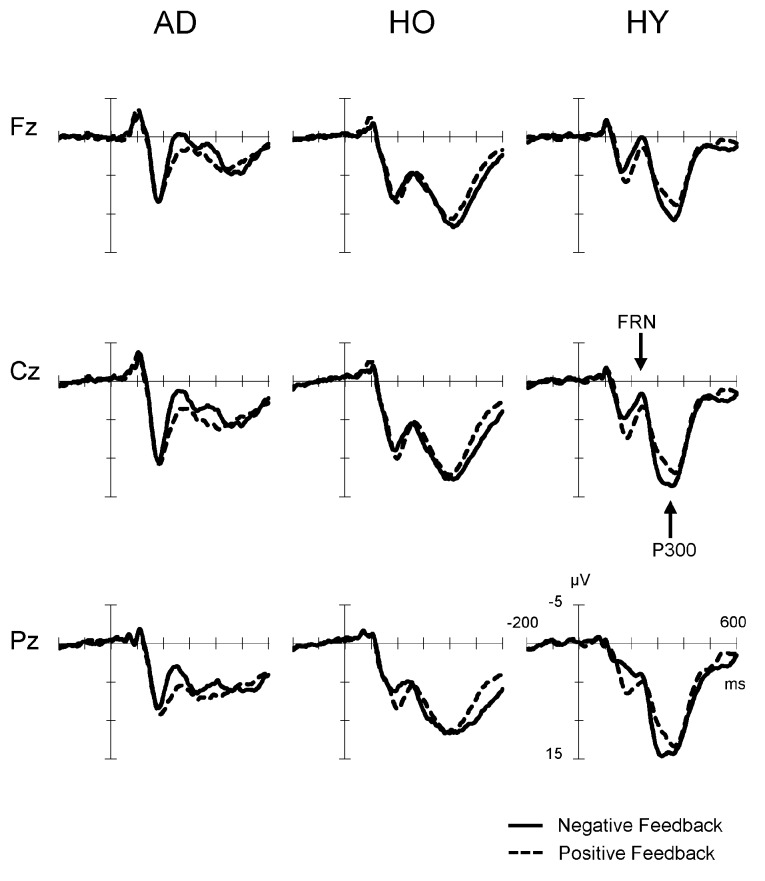
**Grand-average event-related potential (ERP) waveforms time-locked to feedback onset according to conditions (negative feedback or positive feedback) at Fz, Cz and Pz in each group**. Waveforms for negative feedback (solid line) and positive feedback (dashed line) are inserted as overlays. AD, Alzheimer’s disease; HO, healthy old; HY, healthy young.

Figure 2 presents the feedback-locked ERP waveforms at the three midline sites, Fz, Cz and Pz, for positive and negative conditions in each group. In both conditions, FRN appeared at a latency range of 200–400 ms, and P300 appeared at a latency of 300–500 ms. A difference in FRN amplitude between the positive and negative conditions was observed clearly at Fz, Cz and Pz in AD. The FRN difference in HY was also as apparent as that in AD, but not in HO. On the other hand, P300 amplitude was reduced markedly in AD. The P300 amplitude in HY was the largest among the three groups, and P300 in HO was lower than that observed in the HY group.

**Figure 3:**
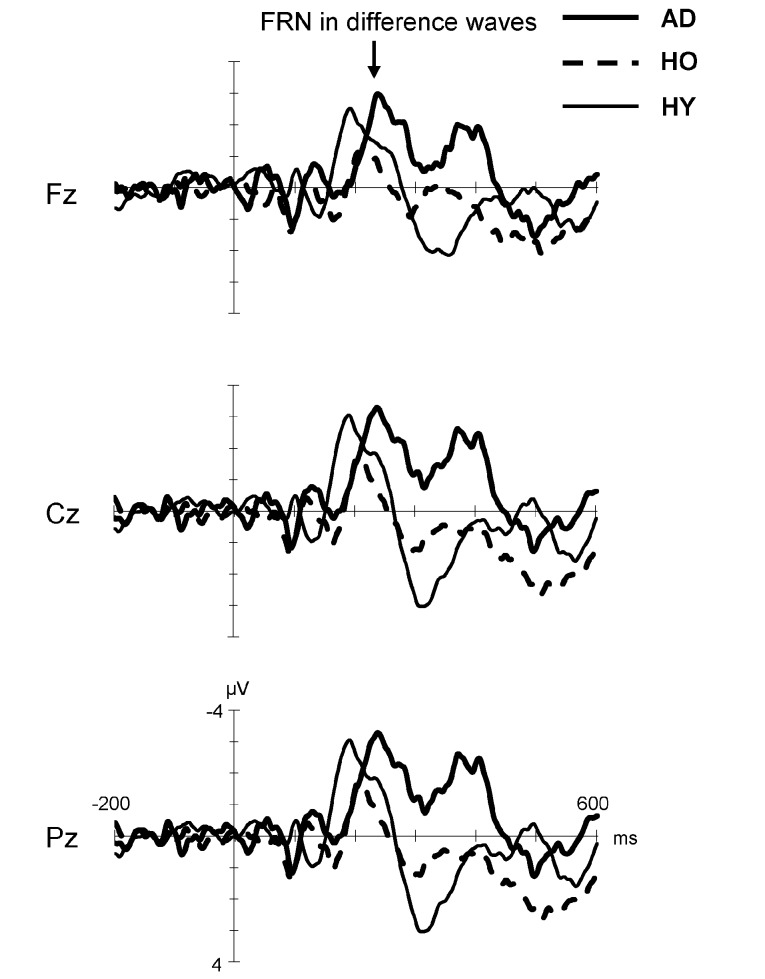
**Grand-average difference waveforms created by subtracting ERP for the positive feedback from that for the negative feedback in each group**. Waveforms for AD (thick solid line), HO (dashed line) and HY (thin solid line) are inserted as overlays. FRN, feedback-related negativity; AD, Alzheimer’s disease; HO, healthy old; HY, healthy young.

Figure 3 displays the difference waves formed by subtracting the ERP for the positive feedback from that for the negative feedback in each group. Here, FRN is the negative deflection in the time window of 200–400 ms after feedback stimuli. The FRN in the AD group showed larger amplitude and prolonged latency compared to that in the HO group. However, the FRN amplitude in HY group was almost the same as that in the AD group, but its latency was shorter than in the latter group. Finally, HO showed smaller FRN amplitude and delayed latency compared to HY.

### FRN in Difference Waves

**Figure 4:**
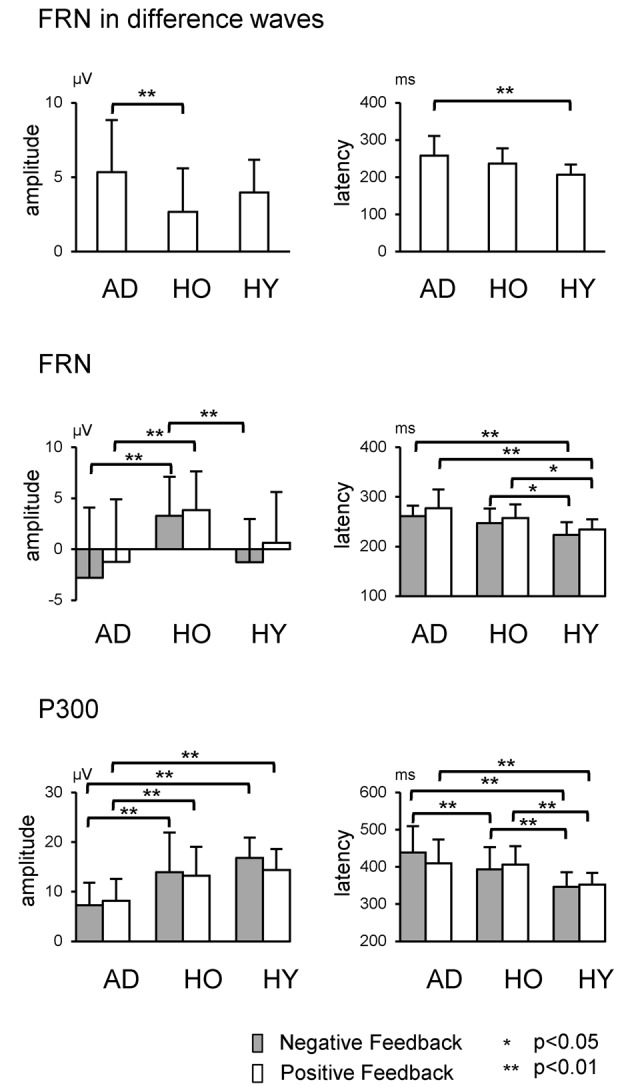
**Each row shows data relating to FRN at Fz and P300 at Pz, respectively**. Error bars denote standard deviations.

The upper row in Figure 4 presents the FRN peak amplitude (left panel) and peak latency (right panel) for the three groups; Table 2 gives the ANOVA results. The ANOVA revealed that the main effect of group for amplitude was significant (*F*_(2,63)_ = 4.1, *ε* = 0.69, *p* = 0.021). *Post hoc* tests indicated the amplitude in AD was significantly larger than that in HO (*p* = 0.015). However, the main effect of channel did not reach significance (*F*_(2,63)_ = 3.0, *ε* = 0.69, *p* = 0.053), nor was interaction of group by channel significant (*F*_(4,126)_ = 1.2, *ε* = 0.69, *p* = 0.296).

**Table 2:** **Repeated two-way ANOVA (group × channel) for feedback-related negativity (FRN) in difference waves data**.

	Main effect of group		Main effect of channel		Interaction (group × channel)	
	*F*	*p*	*F*	*p*	*F*	*p*
FRN peak amplitude	4.1	**0.021**	3.0	**0.053**	1.2	0.296
FRN peak latency	6.3	**0.003**	7.6	**0.001**	0.7	0.602

*Significant p-values are in BOLD*.

Regarding latency, the main effects of group and channel were significant (*F*s_(2,63)_ > 6.3, *ε* = 0.73, *p*s < 0.003). *Post hoc* tests denoted that the latency was prolonged significantly in AD compared to HY (*p* = 0.002), although there was no significant interaction of group by channel (*F*_(4,126)_ = 0.7, *ε* = 0.73, *p* = 0.602).

### FRN

We also examined group differences for the FRN component. The results appear in the middle row of Figure 4 and in Table 3. The ANOVA for the amplitude yielded significant main effects of group at Fz and Cz (*F*s_(2,63)_ > 5.0, *p*s < 0.010). *Post hoc* tests showed that FRN at Fz and Cz were larger for AD compared to HO in both positive and negative conditions (*p*s < 0.028). All channels showed significant main effect of condition (*F*s_(2,63)_ > 17.6, *p*s < 0.001). No significant interaction of group by condition was observed (*F*s_(4,126)_ < 2.9, *p*s > 0.062).

**Table 3:** **Repeated two-way ANOVA (group × condition) for FRN and P300 data**.

	Main effect of group		Main effect condition		Interaction (group × condition)	
	*F*	*p*	*F*	*p*	*F*	*p*
FRN peak amplitude						
Fz	7.6	**0.001**	17.6	**<0.001**	1.6	0.216
Cz	5.0	**0.010**	28.1	**<0.001**	2.9	0.062
Pz	1.3	0.270	44.7	**<0.001**	1.5	0.227
FRN peak latency						
Fz	15.4	**<0.001**	10.9	**0.002**	0.2	0.794
Cz	15.2	**<0.001**	14.9	**<0.001**	0.1	0.975
Pz	12.9	**<0.001**	22.1	**<0.001**	0.1	0.977
P300 peak amplitude						
Fz	11.8	**<0.001**	10.6	**0.002**	2.1	0.129
Cz	10.9	**<0.001**	6.2	**0.016**	4.1	**0.022**
Pz	14.1	**<0.001**	3.0	0.086	4.6	**0.014**
P300 peak latency			
Fz	28.6	**<0.001**	0.9	0.334	3.1	0.053
Cz	16.6	**<0.001**	0.8	0.371	4.0	**0.023**
Pz	13.7	**<0.001**	0.3	0.615	3.3	**0.043**

*Significant p-values are in BOLD*.

For the analysis of latency, significant main effects of group and condition were found at all channels (*F*s_(2,63)_ > 10.9, *p*s < 0.002). *Post hoc* tests indicated that the latency at Fz and Cz were significantly delayed in AD and HO compared to that in HY in both conditions (*p*s < 0.043). There were no significant interactions for group by condition (*F*s_(4,126)_ > 0.2, *p*s > 0.794).

### P300

The analysis for P300 is also depicted in the lower rows of Figure 4 and Table 3. Significant interactions of group by condition were observed at Cz and Pz (*F*s_(4,126)_ > 4.1, *p*s < 0.022). *Post hoc* tests indicated that the amplitudes were significantly smaller in AD than those in HO and HY in both conditions (*p*s < 0.028). There was a tendency for the amplitude to be larger for negative feedback than for positive feedback at Cz and Pz in HY (*p*s < 0.087), while there was no significant difference between the two types of feedback in AD and HO.

With regard to latency, significant interactions of group by condition were observed at Cz and Pz (*F*s_(4,126)_ > 3.3, *p*s < 0.043). *Post hoc* tests indicated that latency was delayed in AD and HO compared with HY in both positive and negative conditions (*p*s < 0.032). Additionally, latency in AD was more prolonged than HO for negative feedback (*p*s < 0.032), but this was not the case for positive feedback.

### Correlation between Neuropsychology and ERP

**Figure 5:**
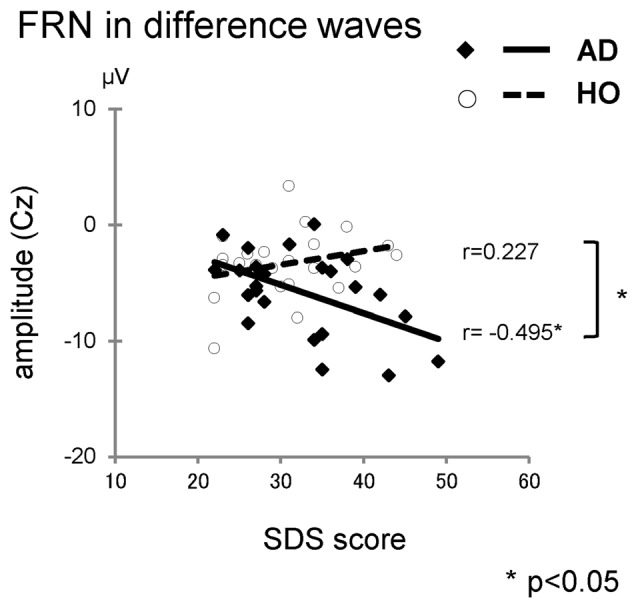
**The correlations between the Self-rating Depression Scale (SDS) and amplitudes of FRN in difference waves**. Diamond and thick solid line: AD, circle and dashed line: HO.

We also performed correlation analyses between the neuropsychological data and the ERP measurements for groups AD and HO (Table 4, Figure 5); and found that there was a significant association between the FRN amplitude in difference waves and SDS at Cz in AD (*r* = −0.495, *p* = 0.023), but not in HO (*r* = 0.227, *p* = 0.309). A direct comparison revealed that the correlation coefficients of the two groups differed significantly (*p* < 0.05). There were significant correlations between FRN amplitude and WFT scores in AD for both conditions (*rs* > 0.46, *p*s < 0.05). P300 amplitudes were positively correlated with WFT scores in both conditions in AD (*p*s < 0.05), and positive feedback in HC (*p*s < 0.01). There were no noticeable correlations between ERP latencies and cognitive function test scores.

**Table 4:** **Correlation coefficients between neuropsychological data and amplitudes of event-related potential (ERP)**.

	FRN in difference waves		FRN				P300			
			Negative		Positive		Negative		Positive	
	AD	HO	AD	HO	AD	HO	AD	HO	AD	HO
MMSE	−0.02	−0.10	0.01	0.15	0.01	0.14	0.03	0.13	0.14	0.16
FAB	0.22	−0.17	0.22	0.10	0.12	0.07	0.01	0.12	−0.05	0.03
WFT	0.23	−0.17	0.53*	0.22	0.46*	0.35	0.59**	0.39	0.45*	0.60**
SDS	−0.50*	0.23	−0.05	−0.23	0.08	−0.24	−0.01	0.07	0.19	−0.18
AS	−0.33	0.01	−0.09	−0.35	−0.01	−0.39	−0.06	−0.04	0.05	−0.20

The results of FRN in difference waves and FRN are in Cz, and P300 in Pz. MMSE, Mini Mental State Examination; FAB, Frontal Assessment Battery; WFT, Word Fluency Test; SDS, Self-rating Depression Scale; AS, Apathy Scale; AD, Alzheimer’s disease; HO, healthy old. *Uncorrected *p* < 0.05, **Bonferoni corrected *p* < 0.05 (=uncorrected *p* < 0.05/6).

## Discussion

The aim of this study was to examine the changes in the monitoring system of AD patients and healthy control participants (HO) during gambling tasks using FRN. Results reveal that the AD group showed a larger amplitude and a delayed latency of FRN compared to the HO group, and the FRN amplitude correlated with cognitive function scores. This study revealed electrophysiological abnormalities of the monitoring function in AD. However, the increased amplitude of FRN in AD was unexpected. It is the opposite of predictions made from the original hypothesis.

The original hypothesis relating to changes in FRN for the AD group was based on prior evidence of aging effects on FRN. Many researchers have reported decreases in FRN amplitude associated with aging (Eppinger et al., 2008; Mathewson et al., 2008; Wild-Wall et al., 2009; Hämmerer et al., 2011; Pietschmann et al., 2011a), and our results also replicated this aging effect. ERN is also known to be affected by aging, with elderly people typically showing smaller ERN than young people (Gehring and Knight, 2000; Falkenstein et al., 2001; Nieuwenhuis et al., 2002). Several research groups have reported that the impairment of error processing and learning in older adults is the result of age-related changes in the mesencephalic dopamine system, such as the loss of dopamine receptors and the deterioration of dopaminergic receptor binding with aging (Volkow et al., 1998; Bäckman et al., 2000; Kaasinen et al., 2000). Based on findings that the efficiency of the dopamine system declines with age, Nieuwenhuis et al. (2002) extended reinforcement learning theory to older adults. They proposed that weakened phasic activity of the midbrain dopamine system leads to reduced negative reinforcement learning signals; implying that elderly people are learning impaired compared to younger adults (Nieuwenhuis et al., 2002). We had expected that this aging effect would be most pronounced in AD, which would mean that AD patients would exhibit smaller amplitudes in monitoring responses. A previous ERN study had indeed reported that amplitudes decreased for AD patients compared to healthy elderly people (Mathalon et al., 2003). However, in contrast to the results of that previous study, we found a significant increase of FRN amplitude in AD patients.

First of all though, whether AD patients in our study could understand the gambling task is a problem when we try to explain this result. Nevertheless, some behavioral studies have reported that AD patients have accomplished more complex tasks like the Iowa Gambling Task (Sinz et al., 2008). In our study, moreover, the switching ratio following the feedback was larger for the negative feedback than the positive feedback in all groups, suggesting that even AD patients could distinguish between positive and negative stimuli, and could avoid the options associated with recent negative results. Therefore, the negative component obtained from the differential waveform in AD could be considered as FRN.

As a result, we demonstrated that AD patients show a higher FRN. It is already known that the source of FRN is located in the ACC (Holroyd and Coles, 2002; Hajcak et al., 2007; Bellebaum and Daum, 2008; Holroyd et al., 2009), and abnormalities of the ACC in AD have been reported at histopathological and physiological levels. In AD patients, accumulation of both beta amyloid and tau (Morishima-Kawashima and Ihara, 2002; Leuba et al., 2009; Murphy and LeVine, 2010; Bloom, 2014), metabolic changes (Bracco et al., 2007; Lim et al., 2012), and decreased blood flows (Dukart et al., 2013; Long et al., 2013; Terada et al., 2013; Lin et al., 2014; Bailly et al., 2015) are observed in the ACC. The ACC is atrophied structurally (Buckner et al., 2005; Jones et al., 2006; Seeley et al., 2009; Krueger et al., 2010), and the anatomical connectivity with other areas is also impaired (Greicius et al., 2004; Rombouts et al., 2005; Wang et al., 2007; Boublay et al., 2016; Hafkemeijer et al., 2016b). On the other hand, higher functional connectivity of the ACC for the salience network has been reported in some resting-state fMRI studies for AD (Zhou et al., 2010; Hafkemeijer et al., 2016a). The salience network plays a crucial role in the detection of salient events from internal and external information (Seeley et al., 2007), therefore the network is inevitably involved in monitoring processing. The enhanced functional connectivity of the ACC in AD is dovetailed with the enhanced FRN in our study in the context of intensified detection of salient feedback information. Thus, the influence of AD pathology on the ACC function might be affected depending on the segregated functional network of the ACC.

One of the possible mechanisms for explaining this discrepancy is the compensation-related utilization of neural circuits hypothesis (CRUNCH; Grady, 2012). The idea of CRUNCH is that more neural resources are recruited by the elderly at low levels of cognitive load (when tasks are easier) than younger adults, who do not need them. At higher load levels, this compensatory mechanism is not effective, leading to less activation in elderly compared with young adults. This could apply to the relationship between AD and the healthy elderly (Grady, 2012). According to CRUNCH, enhanced FRN reflects a compensatory mechanism with larger recruitment of neural activity in AD patients compared to younger adults. Younger adults in our study might search a best strategy or hidden rule to get a better performance although the probabilities of positive and negative feedbacks were truly equal and the sequence was completely random.

Another idea that might explain the discrepancy is obtained from ERP studies of other diseases. A similar discrepancy between FRN and ERN has been reported in several psychiatric diseases in young people (van Meel et al., 2005, 2011; Groen et al., 2008; Holroyd et al., 2008; Vlamings et al., 2008; South et al., 2010; Larson et al., 2011; Santesso et al., 2011). Studies investigating FRN and ERN in children with autism have demonstrated that a robust FRN is equally elicited in children with autism and those with typical development (Larson and Clayson, 2011; Larson et al., 2011; McPartland et al., 2012; Stavropoulos and Carver, 2014), but ERN amplitude is significantly attenuated in autistic children (Vlamings et al., 2008; South et al., 2010; Santesso et al., 2011; Sokhadze et al., 2011, 2012; McMahon and Henderson, 2015). These results imply that individuals with autism may process external and concrete feedback normally, but have difficulty with the internal and more abstract regulation of performance. Similar neurophysiological mechanisms may be operative in AD patients.

As well as our FRN research in AD, there are several reports that have demonstrated FRN enlargement in other diseases. In depressed patients, augmentation of FRN amplitude has been reported (Tucker et al., 2003; Santesso et al., 2008; Cavanagh et al., 2011; Mueller et al., 2015). According to Beck’s cognitive theory of depression (Beck et al., 1979), depressed patients have a “negativity bias”, tending to focus more on negative information. This leads to abnormal responses to negative feedback, and yields an increase of the FRN amplitude. Although our study did not include AD patients with moderate or severe symptoms of depression, there was a significant relationship between FRN amplitude and depression scores only in AD. In AD patients, the “negativity bias” might be aggravated, even in mild depression.

Consistent with previous findings of P300 using an oddball task (Polich et al., 1990; Golob and Starr, 2000; Yamaguchi et al., 2000; Frodl et al., 2002; Bennys et al., 2007; Lee et al., 2013), the present study indicates that P300 exhibits decreased amplitudes and delayed latency in normal aging and AD. As expected, altered P300 was associated with cognitive function scores. The P300 responses are considered to be related to attention and memory processes (Polich, 2007); therefore, lower amplitudes and delayed latency of P300 in AD patients exhibits electrophysiological reflection of attention and memory deficits (Phillips et al., 2004; Bennys et al., 2007; Lai et al., 2010). It is considered that the causes of amplitude reduction in the AD group is atrophy of the hippocampus, decreases in blood flow, and decreases in functional binding. Because similar results were obtained in our gambling task, instead of an oddball task, the gambling task may be a more useful clinical tool in evaluating both FRN and P300 at the same time.

Our study has some limitations. First, we investigated only the alteration of FRN, but not of ERN. It has been speculated that generator sources of FRN and ERN components are located in the anterior cingulate cortex (Gehring et al., 1993; Dehaene et al., 1994; Gehring, 2002; Holroyd and Coles, 2002; Holroyd et al., 2002; Luu et al., 2003), and both potentials are associated with the monitoring system. FRN and ERN have been linked to the monitoring of externally provided and internally generated information, respectively (Müller et al., 2005). Therefore, in order to study abnormalities of the monitoring system in AD, it would be most interesting to examine the changes of both potentials simultaneously. Second, we have not explored any abnormalities of FRN in other cognitive impairment and dementia conditions, i.e., in mild cognitive impairment (MCI), frontotemporal dementia, or dementia with Lewy bodies. It is important to examine FRN changes in MCI, because MCI is a prodromal stage of AD. Several studies have reported that MCI patients show a reduction in P300 amplitude compared to healthy elderly people (Papaliagkas et al., 2008, 2011). Therefore, it is possible that similar ERP changes may occur in MCI, and thus whether FRN amplitude increases in MCI patients should be confirmed.

## Conclusions

In sum, the present study demonstrated that the FRN in AD patients showed larger amplitude and delayed latency compared to age-matched controls, and correlated with depressive tendency. This indicates that enhanced monitoring response in AD patients might reflect a compensatory mechanism and/or negative bias in outcome evaluation. Psychophysiological measures in the feedback process could provide a clue to understand the neurobehavioral changes in AD patients.

## Author Contributions

EN, KO and SY designed the study and wrote the manuscript. EN, FI and RO collected the data. EN and KO performed the analysis. All authors listed, have made substantial, direct and intellectual contribution to the work, and approved it for publication.

## Conflict of Interest Statement

The authors declare that the research was conducted in the absence of any commercial or financial relationships that could be construed as a potential conflict of interest.
